# Can voucher scheme enhance primary care provision for older adults: cross-sectional study in Hong Kong

**DOI:** 10.1186/s12877-020-01851-x

**Published:** 2020-11-03

**Authors:** Johnny T. K. Cheung, Samuel Y.S. Wong, Dicken C. C. Chan, Dexing Zhang, Lawrence H. F. Luk, Patsy Y. K. Chau, Benjamin H. K. Yip, Eric K. P. Lee, Eliza L. Y. Wong, E. K. Yeoh

**Affiliations:** 1 Faculty of Medicine, The Chinese University of Hong Kong, Hong Kong SAR, Hong Kong; 2 JC School of Public Health and Primary Care, The Chinese University of Hong Kong, Hong Kong SAR, Hong Kong

**Keywords:** Incentive, Preventive care, Continuity of care, Public private partnership

## Abstract

**Background:**

The Hong Kong government has launched the Elderly Health Care Voucher (EHCV) scheme to facilitate primary care in the private sector for older adults. This study aimed to examine whether voucher use was associated with a shift of healthcare burden from the public to the private sector, vaccine uptake and continuity of care.

**Methods:**

This cross-sectional survey recruited older adults with ≥3 chronic diseases through convenience sampling from seven general outpatient clinics, seven geriatric day hospitals, and five specialist outpatient clinics of the public healthcare sector in Hong Kong. We used multiple logistic regression to address the study objective.

**Results:**

A total of 1032 patients participated in the survey. We included 714 participants aged 70 or above in the analysis. EHCV use was associated with higher utilization of private primary care services, including general practitioner and family doctor (Adjusted Odds Ratio (AOR) 2.67, 95% Confidence Interval (95%CI) 1.51–4.72) and Chinese medicine clinic (AOR 3.53, 95%CI 1.47–8.49). There were no significant associations of EHCV use with public general outpatient clinic attendance, Accident & Emergency attendance, and hospitalization. Furthermore, EHCV users were more likely to receive pneumococcal vaccination (AOR 2.17, 95%CI 1.22–3.85) and were less likely to visit the same doctors for chronic disease management (AOR 0.10, 95%CI 0.01–0.73).

**Conclusions:**

While the EHCV may promote private primary care utilization and preventive care, older patients continue to rely on public services and the EHCV may worsen continuity of care. Policy-makers should designate voucher usage for chronic disease management and continuity of care.

**Supplementary Information:**

The online version contains supplementary material available at 10.1186/s12877-020-01851-x.

## Background

Ageing is a common risk factor for numerous chronic diseases, such as cancers, diabetes, and cardiovascular diseases [1]. A systematic review reveals that more than half of older adults aged 65 and older are living with multimorbidity, the co-occurrence of two or more chronic diseases [2]. Multimorbidity is associated with a range of adverse outcomes, including frailty and disability, and eventually substantial higher health care utilization and costs [3–6]. In other words, population ageing poses a great challenge to medical service provision and resources [7]. This gives rise to the importance of primary care, the gatekeeper of the whole healthcare system. Family doctor is the major primary care service provider who offers comprehensive, person-centered, continuous, preventive and coordinated care [8]. Indeed, evidence has demonstrated that health systems which rely more on primary care can result in better health outcomes and lower healthcare costs [9, 10]. However, most health systems adopt specialist care or single-disease approaches in the care of patients [11]. As such, we should strengthen primary care to provide coordinated and personalized care to patients with complex healthcare needs [9, 12, 13].

Hong Kong has a two-tier healthcare system in which the service provision is different between the public and the private sector. The public sector provides around 90% of inpatient hospital care. In contrast, only 30% of outpatient services is provided in the public sector, with majority of primary care services provided in the private sector [14]. With a large proportion of medical services offered by the public sector, the Hospital Authority is a statutory body that manages all public hospitals and clinics including general outpatient clinics (GOPCs) and specialist outpatient clinics (SOPCs) in Hong Kong. GOPCs provide care to patients living with chronic diseases with stable conditions and those suffering from mild episodic diseases. If necessary, GOPCs or private family doctors may make referrals to SOPCs for specialist consultation, treatment and investigation. Geriatric Day Hospitals are ambulatory care facilities which provide multidisciplinary assessment, continuous care and rehabilitation to community-dwelling older adults. The government heavily subsidized these public services (over 80%) [15] that patients usually pay at very low prices (Table 1) [16]. For example, fee of each GOPC visit is HKD 50 (USD 6.4), compared to HKD 790 (USD 101.3) for first private outpatient visit. Recipients of the Comprehensive Social Security Assistance and the Higher Old Age Living Allowance can access public healthcare services for free [17]. On the other hand, patients have to pay the charges of private healthcare services with out-of-pocket expenses. Older adults, particularly those economically disadvantaged or living with chronic conditions, tend to attend publicly funded healthcare institutions [18]. The longest waiting time for stable new case booking at SOPCs were 157 weeks for Medicine and 133 weeks for Surgery in the year of 2019/20 [19].

**Table 1:** Charges of public and private healthcare services (in Hong Kong Dollars)

	Public	Private
Accident & Emergency	$180 per visit	–
Inpatient (acute)	$75 admission fee $120 per day	$6650 per day (1st class) $4430 per day (2nd class)
Inpatient (others)	$100 per day	$6120 per day (1st class) $4080 per day (2nd class)
Inpatient medical attendance	–	$680 - $2780 per specialty visit
Outpatient	$50 per visit (general) $135 for first visit, $80 for subsequent visit (specialist)	$790 - $2210 for first visit $640 - $1990 for subsequent visit
Geriatric day hospital	$60 per visit	–

Note:

1 United States Dollar = 7.8 Hong Kong Dollars

The charges were effective on 18 June 2017

Starting from 1 Jun 2018, Higher Old Age Living Allowance recipients who aged 75 or above are waived for charges for public healthcare services

Charges of public outpatient services include medication and consultation fee

The figures were retrieved from website of Hospital Authority of Hong Kong: https://www.ha.org.hk/visitor/ha_visitor_index.asp?Content_ID=10045&Lang=ENG

**Fig. 1:**
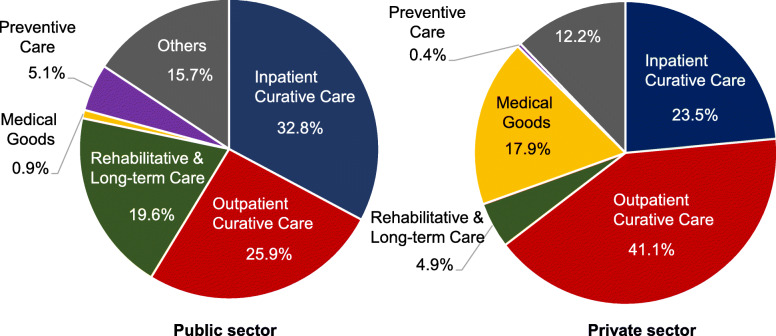
Public and private health expenditure by function in 2016/17. The share attributed to preventive care was small in both the public and private sector. Data were retrieved from the Food and Health Bureau of the Hong Kong Special Administrative Region Government: https://www.fhb.gov.hk/statistics/download/dha/en/table4_1617.pdf

With the increase in demand and costs associated with both population ageing and technological advances [20], there is an urgent need to re-orient healthcare services towards the provision of primary care and preventive services. Nonetheless, the Hong Kong public does not recognize the importance of primary care and disease prevention [21]. As shown in Fig. 1, preventive care shares small proportions of health expenditure in both public and private sectors [20]. In addition, Hong Kong citizens are used to shopping around the private market, rather than developing continuous doctor-patient relationships [21]. While public hospitals and clinics share the same electronic patient record system, most of the individual private healthcare providers keep patient data in paper form. A lack of data sharing often results in fragmented care.

Public-private partnership (PPP) has been adopted by numerous countries to improve health care. Typical examples are infectious disease control programmes in developing regions [22]. HA in Hong Kong has designed several PPP models which purchase services from the private sector in order to enhance patients’ access to healthcare especially chronic condition management. Examples include the Cataract Surgeries Programme, the Colon Assessment PPP, and the Glaucoma PPP [23]. The patients can receive the private services with a fee same as HA one. To promote primary care and the concept of family doctor, the Hong Kong government introduced a PPP programme namely the Elderly Health Care Voucher (EHCV) Scheme in 2009. The scheme offers older adults the voucher as financial incentives to choose private primary healthcare services that meet their needs, including preventive care [24]. A three-year pilot program of the scheme provided five vouchers, each worth HKD 50 (USD 6.4), to older adults aged 70 or above annually. However, the voucher of a limited amount was unsuccessful in encouraging the use of private primary care services [25]. The Hong Kong government has therefore enhanced the annual voucher amount from HKD 250 (USD 32.2) in 2009 to the HKD 2000 (USD 258.0), with a face-value at HKD 1 instead of the original HKD 50 each. The eligible age has been lowered from 70 to 65 since 2017. Recipients can pick over a wide range of healthcare professionals, including general practitioners, family medicine specialists, and Chinese medicine practitioners. The scheme aims to promote preventive care and to develop continuity of care for chronic disease management.

A systematic review shows that voucher programmes could increase healthcare service utilization, improve quality of care, and improve population health outcomes in developing countries [26]. For instances, vouchers improved maternity services in Bangladesh, Cambodia, China and India, family planning services in Kenya, Korea and Taiwan, and sexual and reproductive health care in Nicaragua. The United Kingdom National Health Service also offers an optical voucher to the children under 18 mainly for covering the cost of glasses or contact lenses. However, experience of voucher use in developed world is minimal. There is also no voucher scheme aiming to strengthen primary care services in the rest of the world. Moreover, the EHCV is a community-based voucher which can be used for a wide range of healthcare providers, whereas most other voucher schemes and PPP are service-specific. Research on the EHCV may achieve a new understanding of the voucher use and the PPP models.

Recent local studies revealed that the EHCV in Hong Kong may facilitate the use of private healthcare services, there was no significant reduction in the use of public services [27, 28]. These studies did not assess the impact of the scheme on various aspects of primary care, such as continuity of care and preventive care among older adults with chronic conditions. As a secondary analysis of the Elderly Health Care Quality Survey, this study aimed to examine whether voucher use was associated with (1) a shift of healthcare burden from the public to the private sector, (2) increased utilization of preventive healthcare services, and (3) continuous doctor-patient relationships. These three outcomes are in line with the original goals of the EHCV scheme set by the local government. Our study findings may demonstrate whether a voucher scheme can strengthen primary care in a two-tier healthcare system.

## Methods

### Study design and participant recruitment

We extracted cross-sectional data from the Elderly Health Care Quality Survey (Additional file 1), a part of a large government-commissioned project to evaluate elderly services and end-of-life care in Hong Kong [29]. We recruited older patients with at least three chronic conditions at seven GOPCs, five SOPCs, and seven Geriatric Day Hospitals in the public sector located at all seven clusters of Hong Kong, between June 2016 to July 2017. They were invited to participate in a survey using an interviewer-administered questionnaire. Given that more than 85% of older adults attended public care services for their chronic disease management, we adopted consecutive sampling at the public healthcare institutions to reach the target population until sample size is met. Random sampling is not practical since we did not have access to the patient list in consideration of privacy concerns. With a great number of chronic conditions included, 3+ definition of multimorbidity can provide a greater specificity and is more useful in identifying high-need patients than the 2+ definition [30]. The current study included those aged 70 and above, who were eligible for the EHCV scheme at the time of the survey. We excluded those who were unsure about their voucher use in our analysis.

### Measures

The independent variable of interest (predictor) was the use of EHCV measured by asking participants whether they had ever used the elderly healthcare voucher (yes/no). Dependent variables (outcomes) were grouped in 3 aspects: (1) health service use, (2) preventive care (vaccination history), and (3) continuity of care. First, participants reported whether they had visited the following healthcare facilities in the past 12 months: public GOPCs, public SOPCs, private family medicine clinics, private general practitioner clinics, private Chinese medicine clinics, hospitalization, and Accident and Emergency (A&E) Department. Second, we asked participants whether they had received influenza vaccine during the season of the survey conducted and pneumococcal vaccine. Third, they indicated whether they had visited more than one doctor without referral from doctors for chronic disease management in the past 12 months.

We also collected sociodemographic and health data including age, sex, marital status, education attainment, and self-rated health. We assessed self-rated health on a five-point Likert scale – ‘very good’, ‘good’, ‘fair’, ‘poor’, and ‘very poor’.

### Data analysis

We computed descriptive statistics of the profiles of participants. We performed Pearson’s Chi-square test or Fisher’s exact test (when one or more expected values are less than 5) to compare differences in sociodemographic and health data, and the outcome variables between voucher users and non-voucher users. We conducted multiple logistic regression to explore associations of the voucher use with each of the aforementioned outcome variables, further adjusted for the sociodemographic variables and self-rated health. The analytical framework is similar to that of a previous study, which performed linear regression on association between the ECHV use and the public healthcare service utilization, controlling for demographic, socio-economic and health status characteristics [28]. The covariates were found to be significantly associated with the healthcare usage. In addition, we performed subgroup analyses of the regression to examine potential bias arisen from pooling the data collected at different sites of recruitment (GOPC vs. SOPC vs. Geriatric Day Hospitals).

All the analyses were hypothesis-driven. We used IBM SPSS 24 to perform all the analyses. We reported Adjusted odds ratios (AORs) and 95% confidence intervals (95% CI) and considered a *p*-value < .05 as statistically significant. We adopted likewise deletion which excludes participants from analysis if any single variable is missing. The statistical power is adequate to detect statistical significance from the regression for the variables with missing data.

## Results

A total of 1032 multimorbid older adults aged 60 or above participated in the survey, with a response rate of 44.3% (out of those approached). Among them, 773 were aged 70 or above. Excluding 59 participants who did not indicate whether they had used the EHCV, we included 714 participants in our final analysis. Subgroup analysis by recruitment sites (GOPC, SOPC, and Geriatric Day hospitals) demonstrates that the direction of the associations was more or less consistent across the sites. Thus, we deemed pooling the data collected at different sites appropriate.

Table 2 presents descriptive statistics of the profile of the 714 participants. Among them, 86.6% of participants have used EHCV before. Compared with non-EHCV users, EHCV users were more likely to be older, being female, had visited public SOPC, private general practitioner or family medicine clinic, private Chinese medicine practitioner, and had received pneumococcal vaccine (all with *p* < .05). Public GOPC attendance rate was lower in voucher users than in non-voucher users (73.8% vs. 80.2%), though the statistical difference was not significant (*p* = .178). Meanwhile, EHCV users were less likely to visit the same doctors for chronic diseases (*p* = .011).

**Table 2:** Descriptive characteristics of study population

Characteristics	Overall (*n* = 714)	EHCV users (*n* = 618)	Non-EHCV users (*n* = 96)	*p*-value
	N (%)	N (%)	N (%)	
Age				
70–74	173 (24.2)	127 (20.6)	46 (47.9)	< 0.001
75–79	190 (26.6)	172 (27.8)	18 (18.8)	
80–84	202 (28.3)	183 (29.6)	19 (19.8)	
≥ 85	149 (20.9)	136 (22.0)	13 (13.5)	
Sex				
Male	363 (50.8)	302 (48.9)	61 (63.5)	0.007
Female	351 (49.2)	316 (51.1)	35 (36.5)	
Marital status				
Single/ widowed/ divorced/ separated	256 (36.2)	224 (36.6)	32 (33.3)	0.536
Married/ cohabitation	452 (63.8)	388 (63.4)	64 (66.7)	
Education level				
No schooling	177 (24.9)	161 (26.1)	16 (16.8)	0.083
Primary	274 (38.5)	239 (38.7)	35 (36.8)	
Secondary	200 (28.1)	164 (26.6)	36 (37.9)	
Tertiary	61 (8.6)	53 (8.6)	8 (8.4)	
Self-rated health				
Very Poor/ Poor	136 (19.1)	122 (19.8)	14 (14.6)	0.466
Fair	344 (48.2)	294 (47.6)	50 (52.1)	
Good/ Very good	233 (32.7)	201 (32.6)	32 (33.3)	
Number of chronic conditions				
3	248 (34.7)	212 (34.3)	36 (37.5)	0.682
4	175 (24.5)	156 (25.2)	19 (19.8)	
5	120 (16.8)	102 (16.5)	18 (18.8)	
≥ 6	171 (23.9)	148 (23.9)	23 (24.0)	
Past 12-month health service use				
Public GOPC	533 (74.6)	456 (73.8)	77 (80.2)	0.178
Public SOPC	398 (55.7)	354 (57.3)	44 (45.8)	0.036
Private GP or FM clinic	228 (31.9)	211 (34.1)	17 (17.7)	0.001
Private Chinese medicine clinic	106 (14.8)	100 (16.2)	6 (6.3)	0.011
Hospitalization	312 (44.1)	276 (45.1)	36 (37.9)	0.188
A & E attendance	337 (47.9)	298 (48.9)	39 (41.1)	0.153
Preventive care				
Seasonal Influenza vaccine	353 (50.0)	312 (51.1)	41 (43.2)	0.152
Pneumococcal vaccine	206 (31.6)	189 (33.6)	17 (19.3)	0.008
Care continuity				
Visit same doctor for chronic disease management	626 (91.9)	540 (90.9)	86 (98.9)	0.011

*Abbreviations*: *EHCV* Elderly Healthcare Voucher, *GOPC* General Outpatient Clinic, *SOPC* Specialist Outpatient Clinic, *GP* General Practitioner, *FM* Family Medicine, *A & E* Accident & Emergency

Data were missing for the following variables: Education (*n* = 2), Marital status (n = 6), Self-rated health (n = 1), Hospitalization (n = 7), Accident & Emergency (*n* = 10), Seasonal Influenza vaccine (*n* = 8), Pneumococcal vaccine (*n* = 63), and Visit same doctor for chronic disease management (*n* = 33)

**Fig. 2:**
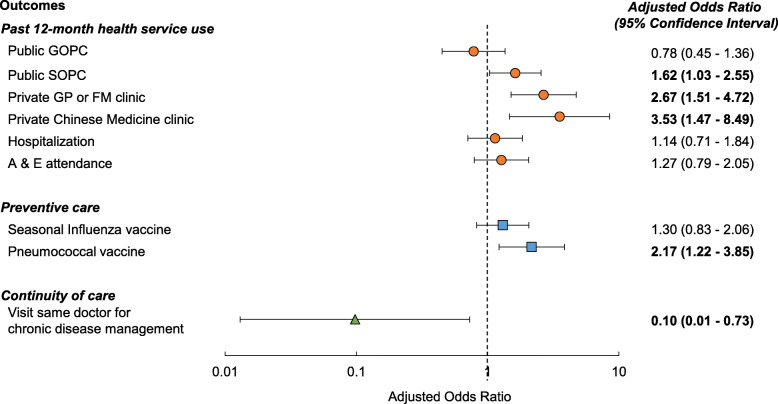
Associations of EHCV use with health service use, preventive care and continuity of care. All models were adjusted for age, sex, education, marital status and self-rated health. Adjusted odds ratios in bold indicate the associations achieve statistical significance at *p* < 0.05. Abbreviations: EHCV = Elderly Healthcare Voucher; GOPC = General Outpatient Clinic; SOPC = Specialist Outpatient Clinic; GP = General Practitioner; FM = Family Medicine; A & E = Accident & Emergency

Figure 2 shows the association of EHCV use with a range of outcomes in multiple logistic regression. The use of EHCV was positively associated with attendance at private primary care services, namely general practitioner or family medicine clinic (AOR 2.67, 95% CI 1.51–4.72) and Chinese medicine clinic (AOR 3.53, 95% CI 1.47–8.49). Associations of EHCV with public healthcare services were not significant, except public specialist outpatient clinic (AOR 1.62, 95% CI 1.03–2.55). For preventive care, EHCV use was significantly associated with higher pneumococcal vaccination uptake (AOR 2.17, 95% CI 1.22–3.85). For continuity of care, EHCV use was associated with a lower odds of visiting the same doctors for chronic disease management (AOR 0.10, 95% CI 0.01–0.73).

## Discussion

The EHCV scheme provides financial incentives to older adults for choosing a range of primary healthcare services in the private sector. On one hand, as expected, voucher use was associated with increased uses of private primary care services (Fig. 2). On the other hand, the EHCV was not significantly associated with public GOPCs, hospitalization, and A&E utilization. Similarly, other local studies reveal that the EHCV was not associated with reduced utilization of public healthcare services and might even encourage dual utilization of public and private healthcare [27, 28]. Descriptive analysis also demonstrates that the utilization rates of public services were much higher than private ones, among both EHCV and non-EHCV users (Table 2). These findings imply that the multimorbid older adults still go for public healthcare service, despite the use of EHCV. This might be attributable to the inadequate monetary amount of voucher (HKD 2000 or USD 258.0) [25], leading to impractically have long-term management for chronic diseases in the private sector.

Ironically, voucher use was associated with higher public SOPC attendance, inconsistent with another local study [28]. One explanation is that with more private family medicine and general practitioner visits by voucher users, more conditions requiring specialist care may have been diagnosed or more referrals for specialist care were made due to health screening. The multimorbid older adults may prefer heavily subsidized SOPCs over self-financed private specialist services (Table 1) [31]. Therefore, the public sector remains the major healthcare providers for older adults with multiple chronic conditions.

In Hong Kong, pneumococcal vaccine is available at a relatively low cost in the private sector due to governmental subsidy, whereas the public sector has not provided free-of-charge pneumococcal vaccine to older adults until 2017/18 [32]. Older adults were likely to receive the vaccine from private healthcare providers and pay for it with the EHCV. This may explain the reason why voucher use was positively associated with pneumococcal vaccination (Fig. 2).

Nonetheless, the use of the EHCV was not significantly associated with seasonal influenza vaccination (Fig. 2). Unlike the pneumococcal vaccine, the influenza vaccine has been freely available for older adults in the public sector at the time of the survey. Hence, older adults might have received the influenza vaccine from public healthcare providers, in order to save the EHCV which is of limited amount for other purposes.

With the financial incentive provided by the EHCV, older adults should have higher purchasing power and thus theoretically more choices of private healthcare service. The quality of private services should be improved through market competition [33]. However, instead of choosing a higher quality of care, we found that patients may end up doctor shopping in the private sector. This may be related to the moral hazard of overutilization, perverse incentives, information asymmetry between patients and healthcare providers, or a lack of a well-established family doctor system in Hong Kong [34]. As shown by our results, EHCV users were less likely to visit the same primary care providers for chronic disease management (Fig. 2). Without a common electronic health record sharing system, the EHCV scheme may have aggravated the fragmentation of clinical information and management approach in the private sector, rather than promoting continuity of care and the concept of family doctor.

While a systematic review shows that all evaluation studies on voucher schemes implemented can improve the health care utilization and quality [26], this is not the case for the EHCV. Given the generic nature of the EHCV scheme, older people can choose over a range of healthcare professionals including optometrists and Chinese medicine practitioners, instead of preventive care or chronic disease management. Moreover, the limited monetary value of the voucher makes it ineffective in inducing changes. An evaluation review suggests a re-design of the EHCV scheme to promote preventive services, chronic disease management, and continuity of care [29]. Specifically, the voucher should be designated for (i) preventive care for early detection and treatment and for (ii) chronic disease management, instead of a broad service coverage under the current scheme. The findings of this study support the suggestions.

### Limitations and further study

Since this study only used secondary data, methodological limitations were related to study design and data availability from the Elderly Health Care Quality Survey. First, we recruited the participants from GOPCs, SOPCs, and geriatric day hospitals in the public sector. We did not include those who have shifted from public to private sector completely due to EHCV incentive. Second, clinical information including disease severity and reasons of consultation was not available in the data source and therefore, we were unable to adjust for any unmeasured confounders or differences in disease severity between voucher users and non-users which may have affected our findings. Third, both predictor and outcomes were binary variables. Fourth, convenience samples have less clear generalizability than probability samples. Future studies should adopt probability sampling and a more detailed record of measurements (interval and ratio), such as the various amount of voucher use and frequency of service use. Further studies should evaluate the effects and cost-effectiveness of the EHCV Scheme.

### Conclusions

This study suggests that the use of the EHCV may be associated with poorer continuity of care without the benefits of reducing the burden of public healthcare service utilization. The findings imply that better private-public partnership model and primary care system are needed in Hong Kong. Our study provides reference for other countries, especially developed ones, in designing voucher schemes for facilitating primary care services use for older patients with multiple chronic diseases.

## Supplementary Information

**Additional file 1.** Elderly Health Care Quality Survey.

## Data Availability

The datasets used and/or analysed during the current study are available from the corresponding author on reasonable request.

## Abbreviations

SOPCs: Specialist Outpatient Clinics
GOPCs: General Outpatient Clinics
EHCV: Elderly Health Care Voucher
A&E: Accident and Emergency
AOR: Adjusted Odds Ratio
95% CI: 95% Confidence Interval
