# Supplementary material for: Can voucher scheme enhance primary care provision for older adults: cross-sectional study in Hong Kong
Source: BMC Geriatr. 2020 Nov 3;20:442. doi: 10.1186/s12877-020-01851-x (PMC7640666; doi:10.1186/s12877-020-01851-x)
Supplement: Supplementary file 1 — Additional file 1. Elderly Health Care Quality Survey. [file 12877_2020_1851_MOESM1_ESM.docx]

**Additional file 1: Elderly Health Care Quality Survey**

1. Have you ever used Elderly Health Care Voucher?

| 1 | Yes | D | Unsure |
| --- | --- | --- | --- |
| 2 | No | N | Aged below 70 |

1. In the past year, did you visit the following health care institutions for chronic disease management and follow-up? (can choose more than one option)

| 1 | Hospital Authority General Outpatient Clinics/ Family Medicine Clinics |
| --- | --- |
| 2 | Hospital Authority Specialist Outpatient Clinics |
| 3 | Private General Practitioner Clinics |
| 4 | Private Family Medicine Clinics |
| 5 | Private Specialist Clinics |
| 6 | Chinese Medicine Clinics |
| 7 | Private Hospital Outpatient Clinics |
| 8 | Geriatric Day Hospitals |
| 9 | Department of Health Elderly Health Centres |
| ) | Others: Please specify: ____________ |
| ! | None |
| D | Don’t know/ Not sure/ Don’t remember |

1. In the past year, did you have hospital admission or Accident and Emergency attendance?

| 1 | Yes, had hospital admission 🡪 Reason: ___________________ |
| --- | --- |
| 2 | Yes, had Accident and Emergency attendance 🡪 Reason: ___________________ |
| 3 | Yes, had hospital admission & Accident and Emergency attendance 🡪 Reason: ___________________ |
| 4 | No |

1. Have you received the following vaccines?
2. Seasonal influenza vaccine (current season)

| 1 | Yes | 2 | No | D | Don’t know/ Not sure/ Don’t remember |
| --- | --- | --- | --- | --- | --- |
| If yes, where did you receive the vaccine? | | | | | |
| 1 | Hospital Authority General Outpatient Clinics | | | 3 | Others: Please specify: ____________ |
| 2 | Private doctor or other private institutions | | |  |  |

1. Pneumococcal vaccine

| 1 | Yes | 2 | No | D | Don’t know/ Not sure/ Don’t remember |
| --- | --- | --- | --- | --- | --- |
| If yes, where did you receive the vaccine? | | | | | |
| 1 | Hospital Authority General Outpatient Clinics | | | 3 | Others: Please specify: ____________ |
| 2 | Private doctor or other private institutions | | |  |  |

1. In the past year, have you visited more than one doctor without referral from other doctors for chronic disease management?

| 1 | Yes | 2 | No | N | N/A |
| --- | --- | --- | --- | --- | --- |
| If yes, what is/are the reason(s): (can choose more than one option) | | | | | |
| 1 | Persistent symptoms | | | 5 | Cannot find the doctor |
| 2 | Distrust in medication | | | 6 | Advice from friends or relatives |
| 3 | Seek another medical advice | | | 7 | Others: Please specify: ____________ |
| 4 | Distrust in the doctor | | |  |  |

1. Overall, in general, would you say that your health is…?

| 1 | Very good | 3 | Fair | 5 | Very poor |
| --- | --- | --- | --- | --- | --- |
| 2 | Good | 4 | Poor |  |  |

Personal characteristics:

1. Gender

| 1 | Male | 2 | Female |
| --- | --- | --- | --- |

1. Education

| 1 | No schooling | 3 | Secondary |
| --- | --- | --- | --- |
| 2 | Primary | 4 | Tertiary |

1. Marital Status

| 1 | Single | 3 | Cohabitation | 5 | Divorced |
| --- | --- | --- | --- | --- | --- |
| 2 | Married | 4 | Widowed | 6 | Separated |
